# HIV Infection Among Partners of HIV-Infected Black Men Who Have Sex with Men — North Carolina, 2011–2013

**Published:** 2014-02-07

**Authors:** Philip J. Peters, Cindy Gay, Steve Beagle, Anupama Shankar, William M. Switzer, Lisa B. Hightow-Weidman

**Affiliations:** 1 Division of HIV/AIDS Prevention, National Center for HIV/AIDS, Viral Hepatitis, STD, and TB Prevention, CDC; 2 University of North Carolina at Chapel Hill; 3 North Carolina Department of Health and Human Services

The incidence of human immunodeficiency virus (HIV) infection has significantly increased among black men who have sex with men (MSM) in the United States, and young black MSM have been disproportionately affected (1). HIV-infected black MSM are also less likely to engage in HIV care (2) and achieve viral suppression (3) than MSM of other races/ethnicities. Engaging in care and achieving viral suppression is a multistep process that starts with diagnosis. Diagnosing persons unaware of their HIV status traditionally has been a critical component of HIV partner services, but partner services also provide an important opportunity to reengage HIV-infected partners in medical care. One approach for partner services involves contacting partners of persons with newly diagnosed HIV infection and using sexual and social network and molecular phylogenetic data to improve the continuum of HIV care among black MSM. To evaluate the effectiveness of that approach, results from a prospective partner services study conducted in North Carolina were examined, and one of the partner networks identified through this study was evaluated in depth. Overall, partner services were provided to 30 black, HIV-infected MSM who named 95 sex partners and social contacts, of whom 39 (41%) previously had been diagnosed with HIV infection. The partner network evaluation demonstrated that HIV-infected and HIV-negative partners were frequently in the same network, and that the majority of HIV-infected partners were already aware of their diagnosis but had not achieved viral suppression. Using partner services to ensure that HIV-infected partners are linked to care and treatment might reduce HIV transmission and might improve outcomes along the continuum of care.

Partner services include a broad array of medical (e.g., linkage to HIV medical care and treatment), prevention (e.g., education and counseling to reduce further HIV transmission), and psychosocial services provided to persons diagnosed with HIV infection and their partners. One critical function of partner services is partner notification, a process routinely used in the prevention and control of sexually transmitted diseases (STDs), including HIV. Persons infected with HIV are interviewed to elicit information about their partners (both sexual and needle-sharing) and social contacts who can then be confidentially notified of their possible exposure to or potential risk for HIV infection (4). Partner notification, as conducted by a health department, is a network-based approach to HIV prevention and treatment. Having information about networks with active HIV transmission provides an opportunity to interrupt chains of transmission (5). Contacting partners within a potential HIV transmission network allows public health practitioners to diagnose HIV-infected persons who are unaware of their status, help HIV-infected partners engage or reengage in medical care, and refer at-risk but HIV-negative partners for HIV prevention services.

Screening Targeted Populations to Interrupt Ongoing Chains of HIV Transmission with Enhanced Partner Notification (STOP) is a prospective study evaluating acute HIV infection diagnosis linked to partner services at 12 HIV testing sites in North Carolina; New York, New York; and San Francisco, California (6). Participants were screened with a rapid HIV enzyme immunoassay (IA). Reactive results were confirmed with a Food and Drug Administration (FDA)–approved HIV-1/HIV-2 antibody differentiation assay (Multispot HIV-1/HIV-2 Rapid Test [Multispot], Bio-Rad Laboratories). Specimens that were negative by the rapid IA were screened for acute HIV infection with a fourth-generation combination antigen/antibody IA (Architect HIV Ag/Ab Combo assay, Abbott Diagnostics) and an HIV-1 RNA test (Aptima HIV-1 RNA qualitative assay, Gen-Probe; 80 rapid negative specimens were pooled for this testing). Repeatedly reactive Architect or Aptima results were confirmed with a second HIV-1 RNA test (m2000 RealTime HIV-1 quantitative assay, Abbott Diagnostics). Based on this testing, HIV-infected participants were diagnosed with either 1) acute HIV infection (HIV rapid test negative but HIV-1 RNA detectable); 2) new, established HIV infection (HIV rapid test reactive and not previously diagnosed); or 3) previously diagnosed HIV infection (HIV rapid test reactive but previously diagnosed).

For partner services, HIV-infected participants (index patients) were offered notification services. Contact information was elicited for sex partners from the previous 3 months for index patients with acute HIV infection and the previous 12 months for index patients with established or previously diagnosed HIV infection. In addition, contact information was elicited for social contacts considered by the index patient to be at high risk for HIV infection (i.e., those who would benefit from an HIV test). Health department personnel trained as disease intervention specialists contacted sex partners and social contacts and used Internet-based communication (e.g., e-mail and social network messaging) and text messaging when available. Sex partners and social contacts were offered HIV testing. HIV status was defined as 1) previously diagnosed HIV infection, 2) newly diagnosed HIV infection, 3) HIV-negative (HIV testing during partner services was negative), or 4) HIV status unknown (could not be located or refused testing). HIV polymerase (*pol*) gene sequences of newly diagnosed HIV-infected persons were analyzed with standard phylogenetic techniques (7) to provide further insight into HIV transmission networks. Specifically, persons were considered to form a cluster when their HIV *pol* sequences were genetically very similar (>97% of aligned nucleotides were identical) and there was high statistical support in phylogenetic analyses (bootstrapping >99% and posterior probabilities =1.0) to suggest the sequences were highly related compared with local controls. This analysis was limited to a subset of black MSM tested in the STOP study in North Carolina for whom HIV-1 *pol* sequences were available.

During September 2011–December 2012, partner notification services were provided to 30 black MSM (median age = 23 years) who had a reactive HIV test result and an available HIV-1 *pol* sequence in the STOP study in North Carolina (45 black MSM who had a reactive HIV test result in the STOP study, but without an HIV-1 *pol* sequence, were excluded from this analysis). The 30 index patients named 95 persons (74 sex partners and 21 social contacts), of whom 39 (41%) previously had been diagnosed with HIV infection, including 14 who had been diagnosed within the most recent year and 17 who were aged <25 years. An additional 29 (31%) of the 95 named sex partners and social contacts accepted an HIV test, and two sex partners (7% of tested and 3% of all sex partners) were newly diagnosed with HIV infection. Of the remaining sex partners and social contacts, eight refused HIV testing, eight refused any partner services counseling, and 11 could not be located. Most sex partners and social contacts were male (98%) and black (81%), with a median age of 26 years (Table). Sex partners were not more likely to be HIV-infected compared with social contacts (p=0.49), and regular (defined as having sex at least weekly) sex partners were not more likely to be HIV-infected compared with nonregular (having sex less than weekly) sex partners (p=0.16). Considering sex partners only, 18 (60%) of the 30 index patients had at least one HIV-infected sex partner identified, and 12 of 17 index patients who named more than one sex partner had both HIV-infected and HIV-negative sex partners.

HIV *pol* gene sequences were available for the 30 index patients, but not for their 95 sex partners and social contacts. Although none of the 30 index patients named another index patient as a sex partner, phylogenetic analyses identified four highly supported clusters involving eight (27%) index patients (two men per cluster). The sexual network and molecular phylogenetic data were combined for each of these four clusters. Based on data collected during April 2012–April 2013, the largest of the resulting networks included 23 black MSM connected by 20 sexual relationships, one social contact, and one molecular phylogenetic link (Figure). Overall, 15 (65%) were HIV-infected, six (26%) tested HIV-negative, one refused HIV testing, and one could not be located. A majority of men in this network were young (aged <25 years), but age-disparate sexual relationships were also represented, and the oldest person in the network was named by four persons (no other member of the network was named by more than two persons). Among nine partners with previously diagnosed HIV infection at the time of the investigation, eight (89%) had been diagnosed within the previous 2 years, but only two were in HIV medical care and only one had achieved viral suppression. Partner services facilitated linkage to care for nine of the HIV-infected partners who were out-of-care, and five additional men achieved viral suppression by August 2013, including the person named by four other persons. Of the six HIV-negative men, five had previously been tested for HIV, but only one had been tested within the last year.

What is already known on this topic?

The incidence of human immunodeficiency virus (HIV) infection has significantly increased among black men who have sex with men (MSM) in the United States, and young black MSM have been disproportionately affected. Previous studies have demonstrated that black MSM have risk behaviors similar to MSM of other racial and ethnic groups but are more likely to have an HIV exposure within their sexual network.

What is added by this report?

Among black MSM who received partner services in North Carolina, a high proportion (41%) of sex partners and social contacts had been previously diagnosed with HIV infection, whereas only 2% of partners were newly diagnosed with HIV infection. Based on sexual and social network and molecular phylogenetic data, a representative partner network demonstrated that HIV-infected and HIV-negative partners were frequently in the same network and that the majority of HIV-infected partners were already aware of their diagnosis but had not achieved viral suppression.

What are the implications for public health practice?

Diagnosing persons unaware of their HIV status provides a potential opportunity to reengage HIV-infected partners already aware of their status in medical care. This public health intervention might be particularly important among young black MSM in an HIV transmission network, who are disproportionately affected by new HIV infections and less likely to maintain sustained access to HIV medical care.

## Editorial Note

Partner notification is an important opportunity to diagnose persons who are unaware of their HIV infection. This report illustrates that partner notification can also be an important opportunity to identify and link to care HIV-infected partners who are aware of their diagnosis but have not achieved viral suppression. This public health intervention might be particularly important among young black MSM, who are disproportionately affected by new HIV infections and less likely to maintain sustained access to HIV medical care (3). Among black MSM in this analysis who were diagnosed with HIV infection, HIV-infected sex partners and social contacts were almost 20 times as likely to have previously diagnosed HIV infection as newly diagnosed HIV infection. These persons are particularly important to reengage in care because they are in a network with potential for further HIV transmission.

Previous studies have demonstrated that black MSM have risk behaviors similar to MSM of other racial and ethnic groups (8) but are more likely to have an HIV exposure within their sexual network (9). This study demonstrates this high-risk environment quantitatively and within an illustrative network. This study suggests that HIV-negative men within these networks remain at-risk for HIV infection and could benefit from preventive interventions (e.g., interactive Internet and mobile device educational resources), more frequent HIV testing and partner testing (e.g., every 3–6 months), and referral for HIV preexposure prophylaxis (PrEP) if they meet clinical criteria (10). A substantial proportion (27%) of the 30 black MSM in this report had an HIV *pol* sequence that clustered by molecular phylogenetic analysis with a person who was not reported as a sex partner. This finding is consistent with the named sex partner and social contact characteristics, which were relatively homogeneous with respect to age (predominantly young), race/ethnicity (81% black), and geography (91% from North Carolina). This degree of clustering and homogeneity suggests that sexual networks among black MSM in North Carolina are highly connected, and that HIV prevention efforts targeting persons (e.g., facilitating access to antiretroviral treatment if HIV-infected or PrEP if HIV negative) in a central sexual network location might result in substantial decreases in HIV transmission.

The findings in this report are subject to at least two limitations. First, partner services were provided to men who had been diagnosed at one of three sexually transmitted infection clinics in North Carolina, and results might not be generalizable to all black MSM. In addition, black MSM without an available HIV-1 *pol* sequence were excluded, which might exclude men who are less likely to link to medical care. However, the results are consistent with national statistics demonstrating high rates of incident HIV infections among black MSM (1), and provide context regarding the underlying drivers of HIV transmission and suggest potential interventions to interrupt these transmissions. Second, sexual-social and phylogenetic networks are limited by self-report and the availability of viral sequences. These networks do not, therefore, include all persons involved in a transmission chain or cluster, nor do they indicate the directionality of HIV transmission. This report does, however, demonstrate the high risk for potential future HIV transmissions within these networks and suggests that a partner services intervention to reengage partners with previously diagnosed HIV infection in HIV medical care might be an effective prevention strategy in this setting.

In this prospective evaluation of partner services provided to black MSM in North Carolina, a high proportion of sex partners and social contacts previously had been diagnosed with HIV infection, and a high proportion of networks had both HIV-infected and HIV-negative sex partners. Partner notification might offer an important means to ensure that all HIV-infected partners (new and previously diagnosed) within these HIV transmission networks engage in HIV medical care. Interventions for HIV-infected (e.g., antiretroviral treatment) and HIV-negative (e.g., PrEP) partners could have a substantial impact on transmission within these networks, improving the HIV continuum of care among black MSM and reducing the number of new infections.

## Figures and Tables

**FIGURE:**
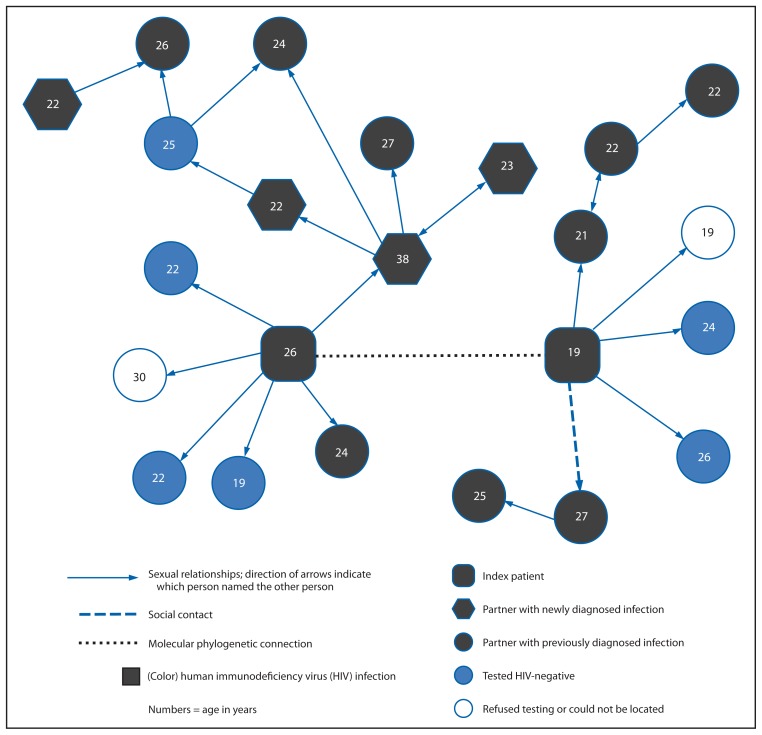
A combined sexual, social, and molecular phylogenetic network of 23 black men who have sex with men, connected by 20 sexual relationships — Screening Targeted Populations to Interrupt Ongoing Chains of HIV Transmission with Enhanced Partner Notification (STOP) study, North Carolina, April 2012–April 2013

**TABLE:** Demographic characteristics of sex partners and social contacts of human immunodeficiency virus (HIV)-infected Screening Targeted Populations to Interrupt Ongoing Chains of HIV Transmission with Enhanced Partner Notification (STOP) study participants, by partner’s HIV status — North Carolina, September 2011–December 2012

	HIV-infected (n = 41*)		HIV-negative (n = 27)		HIV status unknown (n = 27)		Total (n = 95)	
				
Partner characteristics	No.	(%)	No.	(%)	No.	(%)	No.	(%)
**Sex**								
Male	41	(100)	26	(96)	26	(96)	93	(98)
Female	0	—	1	(4)	1	(4)	2	(2)
**Race**								
Black	30	(73)	23	85)	24	(89)	77	(81)
White	8	(20)	3	(11)	2	(7)	13	(14)
Other	3	(7)	1	(4)	1	(4)	5	(5)
**Residence**								
North Carolina	40	(98)	24	(89)	22	(81)	86	(91)
Other state	1	(2)	3	(11)	5	(19)	9	(9)
**Partner type**								
Sex partner	30	(73)	24	(89)	20	(74)	74	(78)
Social contact	11	(27)	3	(11)	7	(26)	21	(22)
**Index case, HIV status**								
Acute HIV infection	6	(15)	7	(26)	4	(15)	17	(18)
Established HIV infection	29	(71)	15	(56)	19	(70)	63	(66)
Previously diagnosed HIV infection	6	(15)	5	(19)	4	(15)	15	(16)
**Frequency of sexual contact** †								
Regular sex partner	9	(22)	4	(15)	3	(11)	16	(17)
Occasional sex partner	4	(10)	7	(26)	6	(22)	17	(18)
Infrequent sex partner	17	(41)	13	(48)	11	(41)	41	(43)
Social contact	11	(27)	3	(11)	7	(26)	21	(22)
**Contact information provided** §								
Internet information only	1	(14)	1	(14)	5	(71)	7	(7)
Address or telephone number	35	(85)	23	(85)	20	(74)	78	(82)
Internet, address, and telephone number	5	(12)	3	(11)	2	(7)	10	(11)

* Includes 39 persons with previously diagnosed HIV infection and two persons with newly diagnosed HIV infection.

† Frequency of sexual contact was defined as regular (had sex at least weekly), occasional (at least monthly but less than weekly), and infrequent (sex less often than monthly or one time only).

§ Information that was provided by the index patient (the participant diagnosed with HIV infection in the STOP study) to allow partner services staff to contact their partners.

## References

[1] Prejean J, Song R, Hernandez A (2011). Estimated HIV incidence in the United States, 2006–2009. PLoS One.

[2] Millett GA, Peterson JL, Flores SA (2012). Comparisons of disparities and risks of HIV infection in black and other men who have sex with men in Canada, UK, and USA: a meta-analysis. Lancet.

[3] Beer L, Oster AM, Mattson CL, Skarbinski J, Medical Monitoring Project (2014). Disparities in HIV transmission risk among HIV-infected black and white men who have sex with men, United States, 2009. AIDS.

[4] CDC (2008). Recommendations for partner services programs for HIV infection, syphilis, gonorrhea, and chlamydial infection. MMWR.

[5] Hogben M, Niccolai LM (2009). Innovations in sexually transmitted disease partner services. Curr Infect Dis Rep.

[6] Pandori MW, Westheimer E, Gay C (2013). The Multispot Rapid HIV-1/HIV-2 differentiation assay is comparable with the Western blot and an immunofluorescence assay at confirming HIV infection in a prospective study in three regions of the United States. J Clin Virol.

[7] Brooks JT, Robbins KE, Youngpairoj AS (2006). Molecular analysis of HIV strains from a cluster of worker infections in the adult film industry, Los Angeles 2004. AIDS.

[8] Millett GA, Flores SA, Peterson JL (2007). Explaining disparities in HIV infection among black and white men who have sex with men: a metaanalysis of HIV risk behaviors. AIDS.

[9] Hurt CB, Beagle S, Leone PA (2012). Investigating a sexual network of black men who have sex with men: implications for transmission and prevention of HIV infection in the United States. J Acquir Immune Defic Syndr.

[10] CDC (2011). Interim guidance: preexposure prophylaxis for the prevention of HIV infection in men who have sex with men. MMWR.

